# Lipidomics Reveals Cerebrospinal-Fluid Signatures of ALS

**DOI:** 10.1038/s41598-017-17389-9

**Published:** 2017-12-15

**Authors:** H. Blasco, C. Veyrat-Durebex, C. Bocca, F. Patin, P. Vourc’h, J. Kouassi Nzoughet, G. Lenaers, C. R. Andres, G. Simard, P. Corcia, P. Reynier

**Affiliations:** 1 Université François-Rabelais, Inserm U930 Tours, France; 2 Laboratoire de Biochimie, CHRU de Tours, France; 3 Institut MITOVASC, UMR CNRS6015-INSERM1083, Université d’Angers, Angers, France; 4 Département de Biochimie et Génétique, CHU d’Angers, France; 5 Centre de Ressources et de Compétences SLA, Service de Neurologie, CHRU Bretonneau, Tours, France; 6 Fédération des CRCSLA Tours et Limoges, LITORALS, Limoges, France

## Abstract

Amyotrophic lateral sclerosis (ALS), the commonest adult-onset motor neuron disorder, is characterized by a survival span of only 2–5 years after onset. Relevant biomarkers or specific metabolic signatures would provide powerful tools for the management of ALS. The main objective of this study was to investigate the cerebrospinal fluid (CSF) lipidomic signature of ALS patients by mass spectrometry to evaluate the diagnostic and predictive values of the profile. We showed that ALS patients (n = 40) displayed a highly significant specific CSF lipidomic signature compared to controls (n = 45). Phosphatidylcholine PC(36:4), higher in ALS patients (p = 0.0003) was the most discriminant molecule, and ceramides and glucosylceramides were also highly relevant. Analysis of targeted lipids in the brain cortex of ALS model mice confirmed the role of some discriminant lipids such as PC. We also obtained good models for predicting the variation of the ALSFRS-r score from the lipidome baseline, with an accuracy of 71% in an independent set of patients. Significant predictions of clinical evolution were found to be correlated to sphingomyelins and triglycerides with long-chain fatty acids. Our study, which shows extensive lipid remodelling in the CSF of ALS patients, provides a new metabolic signature of the disease and its evolution with good predictive performance.

## Introduction

Amyotrophic lateral sclerosis (ALS) is the commonest adult-onset motor neuron disease, characterized by the degeneration of upper and lower motor neurons in the brain and spinal cord. Several reports suggest that ALS is a systemic, heterogeneous disease, and various strategies have been designed for the identification of diagnostic and prognostic biomarkers. Indeed, relevant biomarkers would provide powerful tools for the management of ALS. They would also open new therapeutic avenues, which are currently restricted to the use of riluzole^1^. Some publications have reported instances of protective hyperlipidemia in ALS patients^2^ and increased peripheral lipid clearance in ALS model mice^3^, suggesting that alterations of the lipid metabolism may be involved in the disease.

The brain, the primarily affected organ in ALS, is considered to be quantitatively the richest organ in lipids, comprising cholesterol, phospholipids, sphingolipids and fatty acids (FA). The nature of the lipids and oxidized products from different areas of the brain may thus offer clues to neuronal degeneration, alteration in cell signalling, inflammation, oxidation processes, and the remodelling of membrane structure^4^. The disruption of the lipid organization of the pre-synaptic membrane may affect the structural and physiological properties of the brain, together with the neuronal and synaptic functions, impacting membrane trafficking and the control of protein activity^5^. Polyunsaturated FA are involved in multiple biological pathways, including the synthesis of inflammatory mediators^4^, which have been reported to be involved in the pathophysiology of ALS^6^. The breakdown of cell membranes is a characteristic feature of neuronal degeneration in chronic neurological disorders. Thus, we hypothesized that specific lipid profiles may characterize the pathologic condition of ALS, and we set out to investigate this.

Lipidomics aims at describing and quantifying the complex range of lipid species. Although this “omics” approach has so far been rarely applied to cerebrospinal fluid (CSF), it offers new perspectives in the search for surrogate markers^4,7^. Thus, the investigation of lipid patterns in the CSF may be expected to reveal lipids, specifically released by damaged motor neurons or glial cells, that may help to identify relevant clinical biomarkers of the disease. This would further allow the exploration of certain pathologic mechanisms associated with the deregulation of lipid metabolism and signalling.

## Results

### Patients

The clinical data of 40 ALS patients, and 45 patients with other neurological diseases serving as controls, are listed in Table 1. The sex ratio (Male/Female) was 1:2.4, and the mean age of onset for the ALS patients was 64.67 ± 12.17 years. The site of onset was spinal in 64.1% of the patients, bulbar in 30.8%, and respiratory in the remaining. The median duration of the disease was 33.6 months. No patient was under hypolipemic treatment at the time of data collection. Multivariate analysis between baseline parameters and those recorded one year later showed that three parameters were associated with the duration of ALS, i.e. diagnostic delay (p < 0.001), variation of the ALSFRS-r (revised ALS functional rating scale) score (p < 0.02), and weight loss (p < 0.02).

**Table 1:** Characteristics of ALS patients and controls.

	ALS patients (n = 40)	Control subjects (n = 45)	p-value
	Mean +/− SD or percentage	Mean +/− SD or percentage	
Gender (% female)	42.50	51.10	0.51
Age at sample collection	66.12 +/− 12.10	60.58 +/− 14.01	0.06
Age at onset (years)	64.67 +/− 12.17		
BMI (kg/m2)			
at diagnosis	24.52 +/− 3.82	25.31 +/− 3.20	0.21
at 12 months	25.36 +/− 3.67		
Weight loss at diagnosis (%)	4.09 +/− 7.30		
Diagnosis delay (months)	12.52 + /−9.90		
Site at onset (%)			
Spinal	64.1		
Bulbar	30.8		
Respiratory	5.10		
ALSFRS-r score			
at diagnosis	40.18 +/− 5.25		
at 12 months	32.78 +/− 6.63		
FVC (%)			
at diagnosis	91.72 +/− 26.40		
at 12 months	80.34 +/− 22.16		
Disease duration (months)	33.62 +/− 18.40		

### Lipid profiles

We detected approximately 200 lipids partitioned in 11 classes. Following the pre-processing and processing of data, the data matrix was restricted to 122 lipids. In the CSF lipidome we found glycerophospholipids, i.e. phosphatidylcholine (PC), phosphatidylethanolamine (PE), phosphatidylinositol (PI), and monoetherphosphatidylcholin (MePC); sphingolipids, i.e. ceramide (Cer), and sphingomyelin (SM); glucosylceramide (CerG1), esterified cholesterol (ChE), sterols (ST) and triglycerides (TG). The lipid nomenclature is explained in Supplementary Results, and the lipids used for the statistical analyses are listed in Supplementary Table S1.

### Compared to patients with other neurological diseases, ALS patients carry a specific lipidomic CSF signature

**Figure 1:**
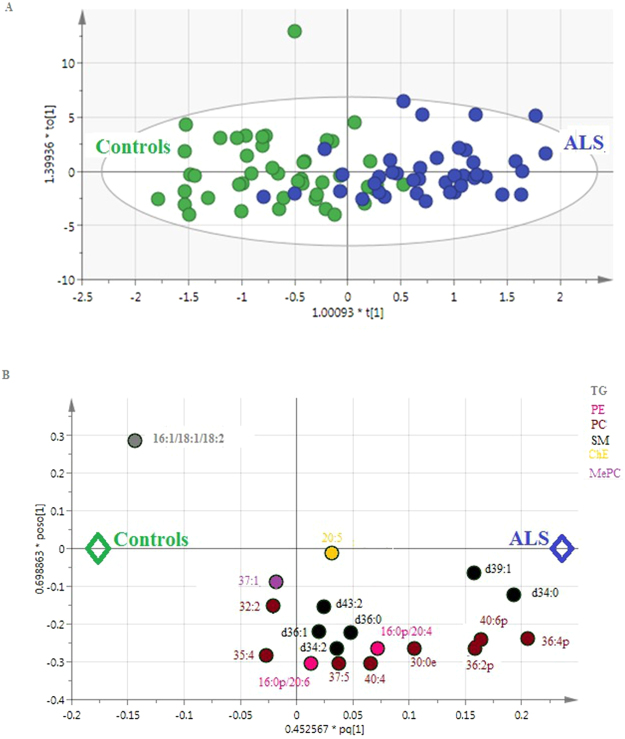
Multivariate analysis (OPLS-DA) of CSF lipids in patients with ALS and controls (n = 85) based on 19 lipids (p = 0.005). (**A**) Score scatter plot. Blue dots: patients with ALS; green dots: controls. X-axis and Y-axis represent score vectors summarizing all the variables entering the analysis: t1 and to1; *R* ^2^ *X*(cum) = 86.8% *R* ^2^ *Y*(cum) = 59.0%, *Q* ^2^(cum) = 0.402 (**B**) Loading scatter plot. Variables near each other are positively correlated; variables opposite to each other are negatively correlated. Variables closer to dots corresponding to “ALS” or “Controls” dots (i.e. with the largest absolute loading values) are higher in the corresponding populations. Lipids from the same family are represented with the same color.

From the OPLS-DA (Orthogonal partial least-squares discriminant analysis) model created from the entire cohort by SIMCA®, 19 lipids appear to be responsible for the discrimination between groups **(**p-CV ANOVA = 0.005, Fig. 1A and B **)**. The model interpreted approximately 86.8% of the total variation in lipids (*R* ^2^ *X*(cum)), and 59.0% of the variations in the various samples (*R* ^2^ *Y*(cum)).

The *biosigner* analysis provided a fitting model (accuracy at 65% in the test set), with the following performances in the test set: mean sensitivity: 64%, specificity: 65.7%, positive predictive value (PPV): 63%, negative predictive value (NPV): 68.5% for the discriminant lipids: PC (Phosphatidylcholine)(36:4p) and PC(36:4e) from RF (Random Forest) algorithms, PC(36:4p), and SM (Sphingomyelin) (d43:2) by the PLS-DA.

**Figure 2:**
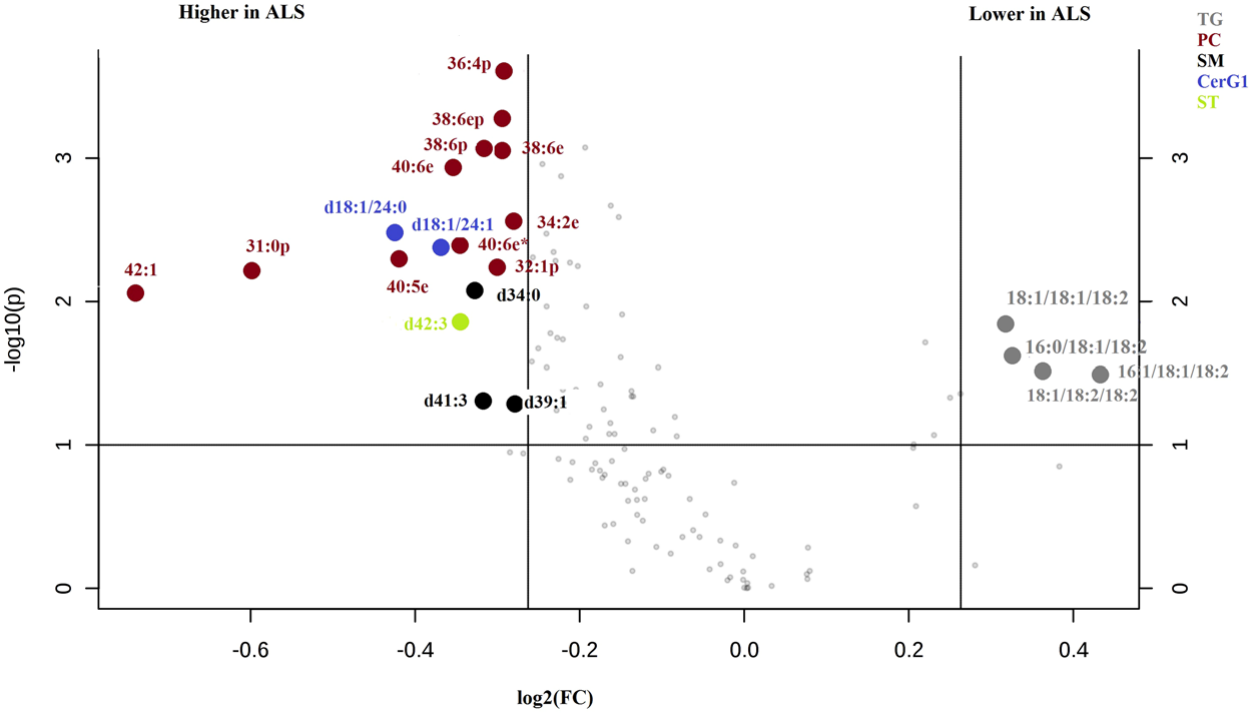
Volcano plot (Metaboanalyst) of the CSF lipids of ALS patients and controls (n = 85), revealing 21 lipids with a p-value < 0.1 and a fold change >1.2. Lipids from the same family are represented with the same color.X-axis corresponds to log2(Fold Change) and Y-axis to −log10(p-value).

Univariate analysis detected 21 lipids with a p-value < 0.1 and a fold change > 1.2 (Fig. 2), and 22 lipids were statistically significant after the Benjamini-Hochberg correction (Supplementary Table S1).

The Venn diagram built from univariate and multivariate analysis is illustrated on Supplementary Figure S1. Among the compounds of the OPLS-DA model, we found six other discriminant metabolites using the volcano plot or univariate analysis after the Benjamini-Hochberg correction, i.e., PC(36:2p), PC(36:4p), PC(40:6p), SM(d34:0), and SM(d39:1), all five being higher in ALS patients, and TG (Triglyceride) (16:1/18:1/18:2), which was lower in ALS patients than in controls. It is worth noting the higher level of PC(36:4p and 36:4e) was the strongest discriminant factor identified by all the statistical approaches used.

### Discriminant lipids common to the brain of ALS model mice and the CSF of ALS patients

Multivariate re-analysis using SIMCA® and focusing on lipids previously quantified in the brain of ALS model mice offered an excellent model for discriminating between ALS and WT mice (p-CV ANOVA = 1.39 × 10^−6^). The *biosigner* analysis also revealed an excellent model with lysoPC (C18:2), as well as 10 PCs and 3 SMs. Univariate analysis showed that lysoPC (C18:2) levels were significantly higher in ALS model mice compared to WT mice (p = 0.00022). The compounds, common to ALS model mice and ALS patients, as detected by the two multivariate findings (SIMCA® and *biosigner*), are represented in Table 2. In particular, we identified PC(32:2), PC(36:2), PC(36:4), and PC(40:6), most of these at higher levels in ALS patients. We also noted some lysoPC in mice which share a common structure with some PCs cited above **(**Table 2 **)**.

**Table 2:** Common lipids between multivariate analysis (SIMCA® and *biosigner* analysis) performed on ALS patients and mice models of ALS. Lipids in bold: identified in patients and mice, and in italic: structures found in lipids common to patients and mice.

Lipid groups	ALS patients	ALS model mice	Examples of common structures
LysoPC		*C16:0*, C17:0, *C18:2*, C20:3, C20:4, C18:1	
PC	C30:0, **C32:2p and e**, C35:4, **C36:2p, C36:4p**, C37:5,C40:4, **C40:6p and e**, MePC(37:1)	C32:0, C32:1, **C32:2p**, C34:1, C34:2, C36:1, **C36:2e**, **C36:4p**, C36:5, C38:0, C38:4, C38:5, C38:6, **C40:6e**	


SM	*C34:2, C34:0, C36:0*, C39:1*, C36:1, C43:2*	*C16:0, C18:0*, *C18:1*, C24:1	

### Lipidomic analysis of CSF at diagnosis predicts the clinical evolution of ALS patients

**Figure 3:**
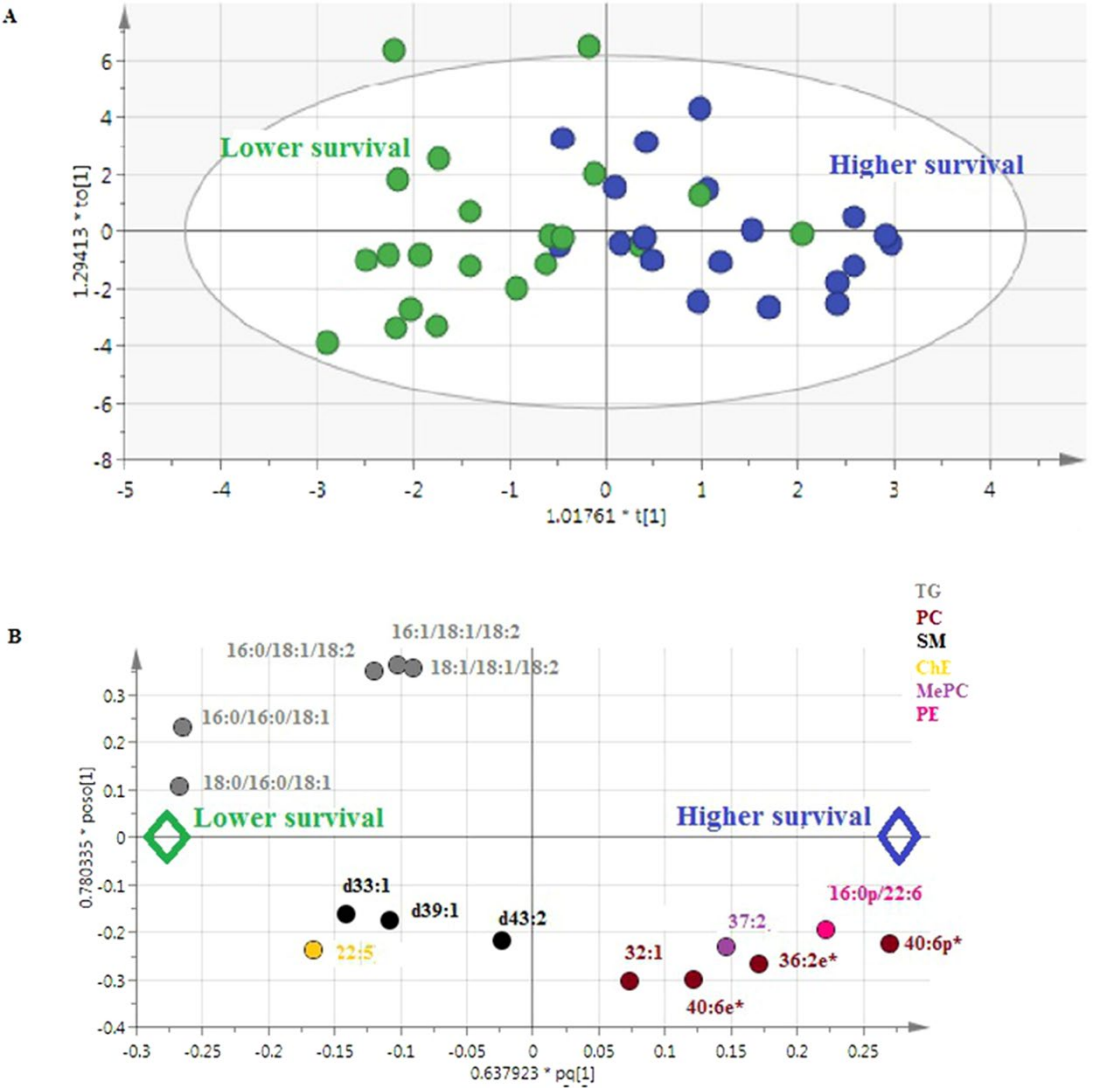
Multivariate analysis (OPLS-DA) of CSF lipids from patients with ALS (n = 40) revealed 16 lipids involved in survival. (**A**) Score scatter plot. Blue dots: ALS patients with survival > median; green dots: ALS patients with survival < median. X-axis and Y-axis represent score vectors summarizing all the variables entering the analysis: t1 and to1 (**B**) Loading scatter plot. Variables near each other are positively correlated; variables opposite to each other are negatively correlated. Variables closer to dots corresponding to “higher survival” or “lower survival” (i.e. with the largest absolute loading values) are higher in the corresponding populations. Lipids from the same family are represented with the same color.

The model predicting the evolution of the ALSFRS-r score, the Forced Vital Capacity (FVC), showed excellent performances using SIMCA® (p-CV ANOVA < 0.002, R^2^X(cum) = 67.7%, R^2^Y(cum) = 83.8%, and Q^2^ = 64.9% from 23 metabolites (p-CV ANOVA = 0.0005), and R^2^X(cum) = 47.4%, R^2^Y(cum) = 75.3%, and Q^2^ = 62% from 16 metabolites (p-CV ANOVA = 0.002), respectively). The model predicting the variation of the BMI showed values of *R* ^2^X(cum) = 52.0%, *R* ^2^Y(cum) = 60.4%, and Q2 = 43.2% from 14 metabolites (p-CV ANOVA = 0.0025) The model predicting the duration of survival (based on median survival) is represented in Fig. 3A and B **(** *R* ^2^X(cum) = 59.4%, *R* ^2^Y(cum) = 51.6%, and Q2 = 41.4% for 15 metabolites (p-CV ANOVA = 0.0007).

**Figure 4:**
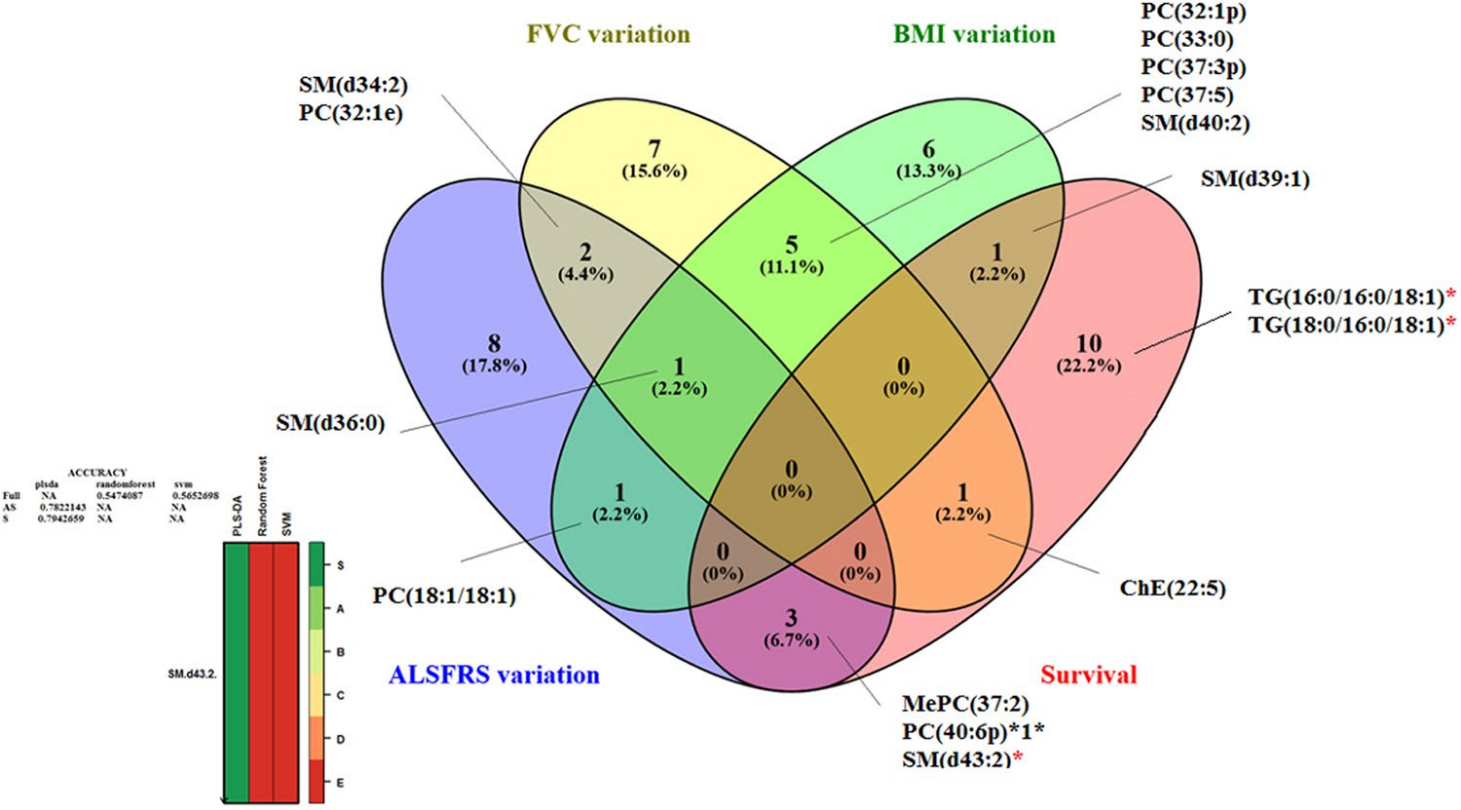
Venn diagram constructed for the lipids highlighted by multivariate analysis (OPLS-DA using SIMCA®) to discriminate between ALS patients according to the modification of ALSFRS-r score, FVC, BMI over one year, and survival. The lipid marked with an asterisk (*), i.e. SM(d43:2), was also highlighted by the *biosigner* analysis, the results of which are shown at the bottom of the figure. The volcano plot shows the lipids with significantly higher levels in cases of ALS, i.e. PC(40:6p) and MePC(Monoetherphosphatidylcholine)(37:2), associated with better survival; SM(d43:2), associated with a lesser decline of the ALSFRS-r score; and SM(d36:0), associated a lesser decline of the FVC. The volcano plot also shows the lipids with significantly lower levels in cases of ALS, i.e. TG(16:0/16:0/18:1) and TG(18:0/16:0/18:1), associated with better survival; and SM(d40:2), SM(d39:1), PC(37:3p) and PC(32:1p), associated with a lesser decline of the BMI.

The *biosigner* analysis revealed an especially robust model (accuracy at 71% in the test set) for predicting the variation of the ALSFRS-r score with the following performances in the test set: mean sensitivity at 62%, specificity at 80.3%, PPV: 79%, NPV at 70.4%. The modelling identified SM(d43:2) as the best discriminant lipid (Fig. 4). Although the *biosigner* analysis did not provide a fitting model for predicting the duration of survival, it highlighted two relevant metabolites: TG(16:0/16:0/18:1) and TG (18:0/16:0/18:1).

The *biosigner* algorithm was used for analyzing the duration of survival from the combination of lipid profile and clinical data. The multivariate modelling confirmed the independent relevance of both biological and clinical findings: we highlighted the same triglycerides (TG(16:0/16:0/18:1), TG (18:0/16:0/18:1)) as previously found, and we also identified diagnostic delay as a discriminating parameter.

After the Benjamini-Hochberg correction, we found a significant association between the higher levels of SM(d43:2) and a lesser decline of the ALSFRS-r score (p = 0.0026).

The Venn diagram (Fig. 4) constructed according to the multivariate models to predict the different parameters of ALS progression highlighted two major lipids: SM(d36:0) and SM(d43:2). Among the lipids highlighted by the Venn diagram in Fig. 4, and also identified by the volcano plot, we observed higher levels of PC(40:6p) and MePC(37:2), together with lower levels of TG(16:0/16:0/18:1) and TG(18:0/16:0/18:1), associated with better survival; lower levels of SM(d40:2), SM(d39:1), PC(37:3p) and PC(32:1p), associated with a lesser decline of the BMI; higher levels of SM(d43:2), associated with a lesser decline of the ALSFRS-r score; and higher levels of SM(d36:0), associated with a lesser decline of FVC.

## Discussion

To our knowledge, this is the first lipidomic study of the CSF of patients with ALS undertaken to identify the profile of lipids likely to help in diagnosing the disease or in predicting its evolution, as well as in exploring the underlying pathologic mechanisms. One of the strengths of our study lies in the combination of several statistical approaches and a validation of models in test sets. We obtained significant models for discriminating between ALS patients and controls and for predicting the variation of the ALSFRS-r score.

### The best discriminant lipids distinguishing ALS patients from controls

The best discriminant lipids distinguishing ALS patients from controls were PC(36:4p), PC(36:4e), and SM(d43:2), with higher levels in cases of ALS, as revealed by the *biosigner* analysis, and SM(d34:0), also with higher levels in cases of ALS, as revealed by both the univariate and the multivariate analyses. Among other discriminant lipids, detected by at least two methods of analysis, we found TG (16:1/18:1/18:2), with lower levels in cases of ALS. According to univariate analysis alone, other PCs were significantly higher in ALS (Supplementary Figure S1). Certain discriminant lipids were also found in the brain of mice, i.e. PC(36:2), PC(36:4), PC(40:6), with higher levels in the ALS groups than in controls **(**Table 2 **)**. This result confirms the quality of our lipidomic approach to the CSF of ALS patients, as well as the pathophysiological relevance of the lipids identified.

The lipids offering the best prognostic capacity for ALS were those highlighted by the *biosigner* analysis, i.e. SM(d43:2), TG (16:0/16:0/18:1) and TG(18:0/16:0/18:1), with higher levels of sphingomyelins (SM) and lower levels of TG associated with a better evolution of the disease.

The involvement of the cholesterol metabolism in ALS, which has been largely reported in pathophysiologic studies of the disease^8^, including a recent publication describing the modification of non-esterified cholesterol in the CSF of ALS patients^9^, was not found in our work. However, our analytical method only detected esterified cholesterol, whereas the non-esterified form that is predominant in the brain^10^, was not measured in our study.

### The important role of ceramides and glucosylceramides in ALS signatures

Our analysis revealed that some sphingolipids, including SM and glucosylceramides (CerG1), were discriminant between ALS patients and controls.

The *biosigner* algorithm identified SM(d43:2) as one of the three discriminating metabolites in the models for predicting the diagnosis and evolution of ALS, stressing the crucial role of the glycosphingolipid pathway in the pathogenesis of the disease. Overall, our results revealing higher levels of SM and CerG1 in the CSF of ALS patients are consistent with those of another study that reported higher levels of ceramides and glucosylceramides in the spinal cord of ALS patients with an associated increase of gluco-cerebrosidase activity^11^. Importantly, the authors suggested that the higher level of glucosylceramide observed was not related to its synthesis but to the decreased expression of the palmitoyltransferase long-chain subunit 2 in ALS motor neurons^12^. It should be noted that ceramides have been associated not only with apoptosis in response to cytotoxic humoral factors^13^, but also with a self-reparative process after injury^14^, and with the synthesis of neurotrophic gangliosides^15^. It has also been suggested that the accumulation of ceramide-derived agents may be protective by reducing ceramide synthesis and increasing the entry of ceramides into the CerG1 pathway, thus limiting the direct toxic effect on motor neurons^11^. Higher GlcCer and downstream glycosphingolipid levels have been also reported in the muscle of ALS model mice as well as in other mice after muscle injury, and GlcCer, Cer and gangliosides were also increased in spinal cord of ALS model mice^16^. These authors also observed the associated upregulation of glucosylceramide synthase in the muscle of ALS model mice and in the CSF of ALS patients. A recent study reports the analysis of some lipids in the CSF of 14 ALS patients (recruited in our ALS center but different from the patients included in this present study) confirming the increased levels of GlcCer and GM1a compared to controls, as in our present study^17^.

### Extensive remodeling of phosphatidylcholines and plasmalogens in ALS

Glycerophospholipids account for more than 50% of the lipid content of membranes, and 45% of the total dry weight of the brain^18^. The discrimination between ALS patients and controls, as well as that between ALS model mice and controls, highlights the essential involvement of phosphatidylcholines, especially of PC(36:4) at higher levels in cases of ALS **(**Table 2). The models predicting the evolution of ALS also revealed the involvement of 7 PCs as shown in Fig. 3. Importantly, PC(32:1p), which is positively associated with ALS and the deleterious progression of the disease, may therefore play a key role in the disease.

A greater concentration of PCs, especially that of PC(16:0/20:4) (i.e. PC(36:4)), may induce a higher metabolic activity of phospholipase A2 (PLA2), resulting in an increased production of lipid mediators, such as eicosanoids that promote inflammation and are generally considered to play a role in the pathophysiology of ALS^19^. Similarly, the release of arachidonic acid (C20:4) and the subsequent generation and accumulation of prostaglandins and lipid peroxides may lead to neurodegenerative processes as observed in Alzheimer’s disease^20^. The identification of the fatty acid chain 22:6 in the discriminant PCs of this model may correspond to the docosahexaenoic acid (DHA) involved in the maintenance of neuronal membranes, signal transduction, neuronal differentiation, neurogenesis, protection against synaptic loss, or spinal cord injury^21^. Whereas some authors have reported an association between the loss of motor neurons in the spinal cord in ALS model mice and the reduction in PC (diacyl-16:0/22:6)^22^, others have highlighted the significant increase of DHA in the frontal cortex of ALS patients^23^.

Plasmalogens (PCs designated as “e”) that represent 22% of the PL in the brain^24^ were highlighted in our study. Plasmalogens, which terminate lipid oxidation and protect ROS-vulnerable myelin, are considered to act as endogenous antioxidants^25,26^. In the context of ALS, plasmalogens may prevent the oxidation of polyunsaturated fatty acids or other lipids.

### Triglycerides with long-chain fatty acids are linked to survival in ALS

Our model discriminating ALS patients from controls highlighted triglycerides (TG): TG(16:1/18:1/18:2), which tend to be decreased in ALS. This observation is consistent with the higher clearance of peripheral triglycerides reported in ALS^3^, and the reduced triglyceride levels in *post mortem* human spinal cord tissue from ALS patients^27^. Two TGs were also identified by *biosigner* analysis predicting disease evolution, with lower levels in patients with a better prognosis. These findings may be related to the role of long-chain FAs in the formation of cytotoxic aggregates of ALS-linked SOD1^28^. The analysis of the structures common to the discriminating lipids observed in ALS patients and ALS model mice revealed lysoPCs containing the FAs: C16:0, C18:0, C18:1 **(**Table 2), as is consistent with the involvement of long-chain FAs.

## Materials and Methods

### Patients

CSF samples were obtained using the same procedure from 40 patients with sporadic ALS at the time of diagnosis and from 45 gender- and age-matched controls. The control group included 45 subjects with the following diagnoses: peripheral neuropathy (n = 12) such as chronic inflammatory demyelinating polyradiculoneuropathy (CIDP) (n = 5), cerebellar ataxia (n = 2), spastic gait paraplegia (n = 3), severe headache (n = 3), multiple sclerosis (n = 3), myasthenia (n = 2), radiculitis (n = 2), walking difficulty (n = 2), the post-polio syndrome (n = 1), myoclonus (n = 1), normal pressure hydrocephalus (n = 1), stroke (n = 1), Parkinson’s disease (n = 1), post-radiotherapy lower motor neuron deficiency (n = 1), facial paralysis (n = 1), syringomyelus (n = 1), myopathy (n = 2), the stiff leg syndrome (n = 1), the CANOMAD syndrome (chronic ataxic neuropathy, ophthalmoplegia, monoclonal IgM protein, cold agglutinins and disialosyl antibodies) (n = 1), multifocal motor neuropathy (MMN) with conduction block (n = 2), paresthesia (n = 1), and progressive supranuclear palsy (n = 1).

The ALS patients satisfied the criteria of a ‘definite’ or ‘probable’ diagnosis defined by the El Escorial workshop^29^. All the participants in this current study gave their informed consent for the use of their CSF for research on biomarkers. The local Ethics Committee of the Centre for Human Research approved the study and the consent process (2016–060). All experiments were performed in accordance with the relevant guidelines and regulations. Information on diagnosis, gender, current age, site of onset, diagnostic delay, and age at onset, was obtained for each patient. We also collected the parameters of disease progression, such as the revised ALS functional rating scale (ALSFRS-r), the forced vital capacity (FVC), and the body mass index (BMI), at the time of diagnosis and again one year later. Moreover, the duration of ALS was defined as the time between the appearance of the first symptoms of the disease and death.

### Lipidomics profile

After the extraction of lipids, lipidomics profiles were performed by high-resolution mass spectrometry (HRMS). *LipidSearch*™ and *TraceFinder*™ version 3.3 software (Thermo Fisher Scientific) were used for the characterization of lipid species.

Briefly, CSF samples were thawed, centrifuged at 3000 g for 5 minutes, aliquoted and conserved at −80 °C prior to the analytical procedure. Lipids were extracted following the method previously reported. Lipid extraction was based on two consecutive treatments with chloroform-methanol (1 mL, v-v: 10-1 then 2-1) on 100 µL of CSF samples mixed with ammonium bicarbonate (170 µL, 155 µM), and 10 µL of an internal standards solution (14:0-16:1-14:0 D5 TG, 16:0 PC-d62, 16:0-d31 Ceramide, 16:0-d31 SM, 16:0-d31-18:1 PE, 16:0-d31-18:1 PI at 10 µg/mL in methanol). The organic phase was collected, evaporated to dryness, and reconstituted with 250 µL of a solution of acetonitrile/isopropanol/water (65/35/5); 10 µL of this preparation were injected for mass spectrometry analysis. Details concerning the mass spectrometry and the validation of the analytical method are given in Supplementary Methods.

### Methodology of data analyses

Univariate and multivariate analyses (using training and test sets) were used to compare the lipidomic profiles of ALS patients with those of controls and to assess different models for predicting disease progression within the ALS group. The four parameters used as markers of disease progression were the modification of the ALSFRS-r score, the BMI, the FVC over a one-year period, and the duration of survival. Venn diagrams were drawn (Venny, version 2.1) to reveal the lipids most significantly associated with the clinical status or the criteria of disease progression in ALS patients.

### Statistical analysis

#### Supervised multivariate analysis

Lipidomic data were analyzed using a multivariate approach with SIMCA® version 13.0 (Umetrics, Umeå, Sweden). Orthogonal partial least-squares discriminant analysis (OPLS-DA) or PLS-DA was performed according the type of variables considered. OPLS-DA evaluated variations in peaks areas between groups: variation in the measured data was partitioned into 2 blocks by the program, one containing variations that correlated with the class identifier and the other containing variations that were orthogonal to the first block and thus did not contribute to discrimination between groups. The OPLS-DA or PLS-DA models were cross-validated by withholding one-seventh of the samples in seven simulations (each sample being omitted once) to avoid over-fitting. VIP values represent the importance of this variable for the OPLS-DA or PLS-DA models, and the loadings characterize the relation between the Y and X variables (lipids). We generated a loading plot that summarizes the most important variables in the separation (p(corr)[1] < 0 indicates the variables associated with one group, and p(corr)[1] > 0 indicates the variables associated with the second group). The quality of the models was described by the cumulative modeled variation in the X matrix *R* ^2^X(cum), the cumulative modeled variation in the Y matrix *R* ^2^Y(cum), and the cross validated predictive ability *Q* ^2^(cum) values. Models were rejected if there was a complete overlap of *Q* ^2^ distributions (*Q* ^2^(cum)^ < ^0) or low classification rates (*Q* ^2^(cum) < 0.05 and eigenvalues > 2). We considered a model robust if *Q* ^2^ > 40% and *R* ^2^ > 50%, but these cut-off values need to be confirmed under biological conditions. CV-ANOVA (ANalysis Of Variance) used for testing cross-validated predictive residuals, is also a diagnostic tool for assessing the reliability of the models. The set of multiple models resulting from the cross validation was used to calculate jack-knife uncertainty measures. We fixed the maximum number of iterations at 200 to ensure convergence of the OPLS algorithm.

According to these parameters, we optimized the model by excluding variables so as to obtain the most relevant model from the minimal number of variables. Thus, we retained the most discriminant lipids based on the VIP with the loading values scaled as correlation coefficients (pcorr).

#### Building a predictive model by combined machine-learning approaches

We used the *biosigner* algorithm in R^30^ to assess a new strategy for discovering significant molecular signatures. *Biosigner* analysis, operating on the same principles as SIMCA®, is complementary and more robust since it is largely more restrictive. This data-mining algorithm is independently wrapped around different machine-learning approaches, i.e. PLS-DA, random forest (RF), and support vector machines (SVM). This *biosigner* strategy aims at finding the smallest pattern that provides a significant model after the combination of sampling (bootstrap), ranking of the VIP, and the evaluation of performance after permutation within the test set and the half-interval search. The final training of the model is based on all samples from the dataset and the selected features. First, the dataset is split into training and testing datasets (by boot-strapping and controlling class proportions). Then, a model is built on the training set, and the performance of prediction is evaluated on the test set. The features are thus rank-based according to their contribution to the model. A feature is considered relevant if the random permutation of the intensities of the other features in the test subsets does not significantly alter the accuracy. Finally, the dataset is restricted to the selected features and the steps detailed above are repeated until the stability of the selected features is obtained. The algorithm returns the tier of each feature for the different classifiers: (1) Tier S corresponds to the lipids that are significant in all steps of the selection; (2) Tier A is significant in all but the last selection; and (3) Tier E regroups all previous rounds of selection.

Importantly, this robust strategy includes bootstraps generating multiple training and test sets, thus providing an independent validation. We have modified the parameters of the *biosigner* algorithm to modulate the size of the training and test sets, the number of bootstraps, and to determine the predictive performance of the models on the independent test set as follows: mean sensitivity, specificity, positive predictive value (PPV), and negative predictive value (NPV). Thus, the performance of the models was determined on test sets independent of the cohorts used to create the models.

We used this algorithm to predict the clinical status and criteria of disease progression in ALS. To predict survival, we used the *biosigner* algorithm on the combination of clinical variables statistically significant after survival analysis and the lipid profile. We used the JMP statistical software, version 7.0.2 (SAS Institute, Cary, NC, USA) to perform survival analysis with the clinical data.

#### Univariate analysis

The univariate analysis of lipid levels was based on fold-change values and the threshold of significance with the volcano plot and the non-parametric Wilcoxon test using Metaboanalyst, version 2.1. We also used the Benjamini-Hochberg correction to highlight the most discriminant lipids after the non-parametric test.

### Comparison of the findings in ALS patients and ALS model mice

Metabolomic profiles of the cerebral cortex of SOD1 G93A (mSOD1) transgenic mice (n = 11) and wild-type (WT) littermates (n = 17) were recently analyzed by our team using a targeted quantitative metabolomics approach^31^. We have now re-analyzed the raw data of the lipids reported in this work using the same statistical analysis as performed on human samples.

## Conclusion

Our lipidomics study, the first of its kind to our knowledge, comparing the lipid profiles of ALS patients to those of controls with various other neurological diseases, revealed specific CSF signatures of ALS, indicative of the extensive remodeling of the lipidome in the disease. The statistical power of our modeling approach revealed the most significant CSF lipids discriminating between ALS patients and controls. These results were consistent with those obtained in the brain cortex of ALS model mice. Some of the discriminant lipids proved to be good predictors of the decline of ALSFRS-r. The precise mechanisms underlying the remodeling of the lipidome remain to be elucidated. However, our findings suggest that further work ALS lipid exploration should focus on three main discriminant classes of lipids, i.e. phosphatidycholines, glycosphingolipids, and long-chain FAs.

## Electronic supplementary material

supplementary information
