# Supplementary material for: Lipidomics Reveals Cerebrospinal-Fluid Signatures of ALS
Source: Sci Rep. 2017 Dec 15;7:17652. doi: 10.1038/s41598-017-17389-9 (PMC5732162; doi:10.1038/s41598-017-17389-9)

**LIPIDOMICS REVEALS CEREBROSPINAL-FLUID SIGNATURES OF ALS**

Blasco H, PharmD, PhD ^1,2,3^ Veyrat-Durebex C, PharmD, PhD ^3,4^ Bocca C,^3,4^ Patin F,^1,2^ Vourc’h P,PhD^1,2^ Kouassi Nzoughet J,PhD^4^ Lenaers G, PhD ^4^ Andres CR,MD, PhD^1,2^ Simard G, PharmD, PhD ^3,5^ Corcia P *,MD, PhD^1,6,7^ Reynier P *, MD, PhD^3,4^

**SUPPLEMENT INFORMATION**

**-Supplementary Methods**

**Liquid chromatography coupled with high-resolution mass spectrometry (LC-HRMS)**

**-Supplementary Results**

**Lipids nomenclature**

**-Supplementary Table S1:** The list of lipids detected in the CSF of ALS patients and controls with a variation coefficient < 30% of that in the quality controls.

-**Supplementary Figure S1**: Venn diagram constructed for the lipids highlighted by univariate analysis (Wilcoxon test by Metaboanalyst *via* the volcano plot, and with the Benjamini-Hochberg correction) and multivariate analysis (OPLS-DA using SIMCA®) to discriminate between patients with ALS and controls (n=85). Lipids marked with an asterisk (*) were also highlighted by the *biosigner* analysis. All the discriminant lipids were at significantly lower levels in cases of ALS than in controls, except for TG (16:1/18:1/18:2).

**Supplementary Methods**

.

**Liquid chromatography coupled with high-resolution mass spectrometry (LC-HRMS)**

LC-HRMS was based on based on the UPLC Ultimate 3000 system (Dionex, Thermo Fisher Scientific, Bremen, Germany) coupled to a Q-Exactive Mass Spectrometer (Thermo Fisher Scientific). A Thermo Scientific Q Exactive mass spectrometer, equipped with electrospray ionization source, was used for this study in positive mode. The HESI source worked with a spray voltage of 3.5 kV, a capillary temperature of 250°C, a heater temperature of 350°C, sheath gas flow of 35 arbitrary units, auxiliary gas flow of 10 arbitrary units and spare gas flow of 1 arbitrary units. During the full scan acquisition, range going from 120 to 1800 m/z, the instrument operated at 70,000 resolutions, with an Automatic Gain Control (AGC) target of 3e6 and a maximum injection time (IT) of 250 ms. During the fragmentation, processed on several QC at the beginning and at the end of the sequence, the isolation window was set at 1 m/z, the instrument operated at 35,000 resolutions, with an Automatic Gain Control (AGC) target of 2e5, a maximum IT of 125 ms and a general NCE of 30eV with a stepped NCE at 50%. Chromatography was carried out using a Dionex UltiMate® 3000 UHPLC equipped with a Phenomenex Kinetex 1.7 µm EVO - C18, 150 mm × 2.10 mm, 100Å HPLC column kept at a temperature of 45°C. A multi-step gradient (preceded by an equilibration time of circa 3 minutes), with a mobile phase A of 0.1 % formic acid and 10 mM of  ammonium formate in ACN/water (60/40) and a mobile phase B in IPA/ACN (90/10) with 0.1% of formic acid and 10 mM of  Ammonium formate, was used with a flow rate maintained at 0.260 ml/min during a runtime of 30 min. The UHPLC autosampler temperature was set at 10°C.

Twelve quality control (QC) samples were injected to equilibrate the system and monitor the reproducibility of the method. We applied the following method to keep only lipids with robust measurement in the final dataset : lipid levels were normalized to the sum of all the lipids measured, and lipids exhibiting more than 30% of variation coefficient in the QC samples were excluded from the dataset used for statistical analyses. The samples were randomized before the pre-analytical step. The experiments were performed by HB who was blinded to the data corresponding to the samples.

**Supplementary Results**

**Lipids nomenclature**

Sphingomyelins (SMs) are a type of sphingolipid consisting of phosphorylcholine and ceramide. Ceramides, i.e. N-acylsphingosines, are by-products of sphingomyelin hydrolysis and may also be synthesized from serine and palmitate. For example, glucosylceramides that contain one or more sialic acids linked to the sugar chain, play key roles in the biosynthesis of glycosphingolipids. The nomenclature of these lipids includes the total number of carbons and unsaturation, with “d” designating the shorthand notation of sphingolipids with 1,3 dihydroxy long-chain bases.

Phosphatidylcholines (PC) are phospholipids composed of a choline head group and glycerophosphoric acid, with FAs of varying lengths and saturations. Exact FA composition may not be identified by our lipidomics approach but the analysis provides the total number of carbons and the number of unsaturations. For example, PC(36:4) corresponds to a PC with 36 carbons and 4 unsaturations on the 2 FAs. The letters e and p, as in PC(36:4e) or PC(36:4p), correspond to ether and vinyl ether bonds, respectively. The [ether phospholipid](https://en.wikipedia.org/wiki/Ether_phospholipid)s characterized by the presence of a vinyl ether linkage at the sn-1 position and an ester linkage at the sn-2 position are designated as plasmalogens that represent up to 20% of the total phospholipid mass in humans. The sn-1 position is often derived from C16:0, C18:0, or C18:1 fatty alcohols and the sn-2 position corresponds to polyunsaturated [fatty acids](https://en.wikipedia.org/wiki/Fatty_acid).

Triglycerides (TG) are triacylglycerides with each molecule of glycerol esterified with three FAs.

| **Supplementary Table S1:** The list of lipids detected in the CSF of ALS patients and controls with a variation coefficient < 30% of that in the quality controls.  The mean, standard deviation(SD) and p-value corresponding to the comparison of lipid levels using the Wilcoxon test are shown.  Lipids in bold type correspond to those statistically significant after the Benjamini-Hochberg correction. | | | | | |  |
| --- | --- | --- | --- | --- | --- | --- |
|  |
|  | **ALS patients** | | **Control subjects** | | **p-value** | **Fold Change (ALS/Controls)** |
|  | **Mean** | **SD** | **Mean** | **SD** |
| **PC(36:4p)** | 0.00371 | 0.00092 | 0.00307 | 0.00076 | **0.00032** | 1.21 |
| **PC(36:4e)** | 0.00257 | 0.00055 | 0.00225 | 0.00087 | **0.00099** | 1.14 |
| **PC(38:6e)** | 0.00129 | 0.00035 | 0.00104 | 0.00037 | **0.00106** | 1.23 |
| **PC(38:6p)** | 0.00045 | 0.00013 | 0.00036 | 0.00013 | **0.00106** | 1.24 |
| **PC(32:1e) *1*** | 0.00193 | 0.00055 | 0.00163 | 0.00042 | **0.00127** | 1.19 |
| **PC(40:6e)** | 0.00030 | 0.00009 | 0.00023 | 0.00010 | **0.00139** | 1.28 |
| **PC(38:2)** | 0.00153 | 0.00035 | 0.00131 | 0.00034 | **0.00153** | 1.17 |
| **PC(38:4p) *1*** | 0.00223 | 0.00049 | 0.00199 | 0.00111 | **0.00239** | 1.12 |
| **PC(38:4p)** | 0.00223 | 0.00049 | 0.00200 | 0.00116 | **0.00284** | 1.11 |
| **PC(34:2e) *1*** | 0.00951 | 0.00300 | 0.00782 | 0.00264 | **0.00292** | 1.22 |
| **PC(40:6p)** | 0.00035 | 0.00012 | 0.00030 | 0.00020 | **0.00327** | 1.18 |
| **CerG1(d18:1/24:0)** | 0.00042 | 0.00023 | 0.00032 | 0.00017 | **0.00377** | 1.35 |
| **PC(40:6e) *1*** | 0.00030 | 0.00010 | 0.00023 | 0.00011 | **0.00434** | 1.27 |
| **CerG1(d18:1/24:1)** | 0.00072 | 0.00034 | 0.00056 | 0.00028 | **0.00446** | 1.29 |
| **PC(36:2p)** | 0.00029 | 0.00008 | 0.00025 | 0.00012 | **0.00484** | 1.17 |
| **SM(d35:1)** | 0.00085 | 0.00021 | 0.00071 | 0.00019 | **0.00525** | 1.20 |
| **PC(37:4)** | 0.00052 | 0.00013 | 0.00044 | 0.00014 | **0.00555** | 1.17 |
| **SM(d42:3)** | 0.00569 | 0.00133 | 0.00491 | 0.00127 | **0.00570** | 1.16 |
| **PC(34:1e)** | 0.00803 | 0.00198 | 0.00698 | 0.00200 | **0.00601** | 1.15 |
| **PC(32:1p)** | 0.00072 | 0.00027 | 0.00058 | 0.00022 | **0.00618** | 1.23 |
| **PC(40:5e)** | 0.00017 | 0.00008 | 0.00013 | 0.00007 | **0.00824** | 1.34 |
| SM(d34:0) | 0.00431 | 0.00153 | 0.00343 | 0.00139 | 0.00826 | 1.25 |
| PC(31:0p) | 0.00005 | 0.00003 | 0.00003 | 0.00002 | 0.00987 | 1.51 |
| SM(d18:1/24:1) | 0.00983 | 0.00234 | 0.00860 | 0.00219 | 0.01122 | 1.14 |
| SM(d18:2/24:2) | 0.00051 | 0.00015 | 0.00044 | 0.00015 | 0.01122 | 1.18 |
| PC(32:0e) | 0.00167 | 0.00033 | 0.00151 | 0.00040 | 0.01271 | 1.11 |
| ST(d42:3) | 0.00013 | 0.00006 | 0.00010 | 0.00006 | 0.01473 | 1.28 |
| TG(18:1/18:1/18:2) | 0.01028 | 0.00824 | 0.01281 | 0.00824 | 0.01473 | 0.80 |
| PC(36:2e) *1* | 0.00092 | 0.00023 | 0.00078 | 0.00026 | 0.01703 | 1.18 |
| PC(36:2e) | 0.00115 | 0.00029 | 0.00098 | 0.00033 | 0.01829 | 1.17 |
| PC(32:1e) | 0.00095 | 0.00029 | 0.00082 | 0.00033 | 0.01873 | 1.17 |
| TG(18:1/18:1/18:1) | 0.02123 | 0.01746 | 0.02473 | 0.01411 | 0.01964 | 0.86 |
| SM(d41:2) | 0.00086 | 0.00027 | 0.00073 | 0.00024 | 0.02156 | 1.19 |
| PC(35:1) | 0.00286 | 0.00055 | 0.00257 | 0.00062 | 0.02476 | 1.11 |
| TG(16:0/18:1/18:2) | 0.02705 | 0.02159 | 0.03386 | 0.02133 | 0.02476 | 0.80 |
| SM(d41:1) | 0.00104 | 0.00034 | 0.00087 | 0.00025 | 0.02650 | 1.20 |
| PC(20:0e/18:4) | 0.00061 | 0.00018 | 0.00051 | 0.00019 | 0.02900 | 1.18 |
| SM(d33:1) | 0.00086 | 0.00014 | 0.00080 | 0.00019 | 0.02900 | 1.08 |
| TG(18:1/18:2/18:2) | 0.00482 | 0.00290 | 0.00619 | 0.00394 | 0.02965 | 0.78 |
| TG(16:1/18:1/18:2) | 0.00835 | 0.00504 | 0.01127 | 0.00724 | 0.03240 | 0.74 |
| SM(d42:1) | 0.00202 | 0.00066 | 0.00179 | 0.00060 | 0.03772 | 1.13 |
| PC(35:2) | 0.00096 | 0.00024 | 0.00083 | 0.00027 | 0.04109 | 1.15 |
| PC(34:2) | 0.02630 | 0.00580 | 0.02391 | 0.00595 | 0.04197 | 1.10 |
| TG(18:0/18:1/20:4) | 0.00134 | 0.00124 | 0.00161 | 0.00103 | 0.04378 | 0.83 |
| PC(32:1) | 0.01950 | 0.00411 | 0.01775 | 0.00447 | 0.04565 | 1.10 |
| PC(36:4) | 0.03202 | 0.00640 | 0.02912 | 0.00784 | 0.04565 | 1.10 |
| PC(40:3p) | 0.00011 | 0.00005 | 0.00009 | 0.00006 | 0.04570 | 1.16 |
| TG(16:0/18:1/18:1) | 0.05127 | 0.03807 | 0.06097 | 0.03343 | 0.04661 | 0.84 |
| PC(34:2e) | 0.00073 | 0.00021 | 0.00064 | 0.00025 | 0.05063 | 1.15 |
| SM(d41:3) | 0.00009 | 0.00004 | 0.00007 | 0.00004 | 0.05168 | 1.21 |
| SM(d39:1) | 0.00054 | 0.00020 | 0.00045 | 0.00014 | 0.05168 | 1.24 |
| SM(d40:1) | 0.00313 | 0.00088 | 0.00278 | 0.00068 | 0.05606 | 1.13 |
| SM(d34:1) | 0.02206 | 0.00291 | 0.02081 | 0.00411 | 0.06320 | 1.06 |
| SM(d38:1) | 0.00348 | 0.00091 | 0.00311 | 0.00066 | 0.06972 | 1.12 |
| ChE(16:0) | 0.00073 | 0.00023 | 0.00064 | 0.00023 | 0.07389 | 1.14 |
| PC(42:1) | 0.00002 | 0.00002 | 0.00002 | 0.00002 | 0.07435 | 1.67 |
| MePC(35:0) | 0.00396 | 0.00094 | 0.00367 | 0.00114 | 0.07826 | 1.08 |
| PC(22:2/14:3) | 0.00083 | 0.00026 | 0.00074 | 0.00024 | 0.08283 | 1.12 |
| PC(30:0e) | 0.00017 | 0.00005 | 0.00016 | 0.00006 | 0.08283 | 1.12 |
| PC(33:1) *1* | 0.00006 | 0.00002 | 0.00007 | 0.00003 | 0.08440 | 0.85 |
| SM(d36:4) | 0.00110 | 0.00015 | 0.00104 | 0.00023 | 0.08600 | 1.06 |
| PC(40:5) | 0.00145 | 0.00046 | 0.00127 | 0.00037 | 0.08927 | 1.14 |
| TG(45:3) | 0.02883 | 0.02554 | 0.03326 | 0.02014 | 0.09787 | 0.87 |
| PC(37:3p) | 0.00011 | 0.00035 | 0.00012 | 0.00040 | 0.10335 | 0.87 |
| PC(40:7) | 0.00185 | 0.00051 | 0.00167 | 0.00059 | 0.10523 | 1.11 |
| PC(36:2p) *1* | 0.00021 | 0.00010 | 0.00018 | 0.00010 | 0.11301 | 1.20 |
| ChE(20:3) | 0.00227 | 0.00094 | 0.00194 | 0.00079 | 0.12337 | 1.17 |
| SM(d38:2) | 0.00175 | 0.00047 | 0.00157 | 0.00038 | 0.12771 | 1.12 |
| ChE(20:4) | 0.01718 | 0.00678 | 0.01486 | 0.00587 | 0.12993 | 1.16 |
| PC(34:2p) | 0.00077 | 0.00026 | 0.00068 | 0.00025 | 0.13218 | 1.13 |
| TG(16:0/16:0/18:1) | 0.04702 | 0.02036 | 0.06131 | 0.03734 | 0.13910 | 0.77 |
| PC(20:0/18:3) | 0.00439 | 0.00146 | 0.00386 | 0.00121 | 0.14629 | 1.14 |
| PC(27:0/11:4) | 0.02440 | 0.00462 | 0.02279 | 0.00564 | 0.14629 | 1.07 |
| PC(36:3) *1* | 0.01030 | 0.00279 | 0.00912 | 0.00278 | 0.14876 | 1.13 |
| ChE(22:5) | 0.00022 | 0.00012 | 0.00018 | 0.00011 | 0.14932 | 1.22 |
| PC(36:1) | 0.02735 | 0.00577 | 0.02550 | 0.00587 | 0.15125 | 1.07 |
| PC(18:1/18:1) | 0.02745 | 0.00498 | 0.02533 | 0.00525 | 0.15633 | 1.08 |
| MePC(37:2) | 0.00131 | 0.00039 | 0.00116 | 0.00045 | 0.15892 | 1.12 |
| PC(33:1) | 0.00238 | 0.00044 | 0.00223 | 0.00049 | 0.16154 | 1.07 |
| SM(d40:2) | 0.00212 | 0.00052 | 0.00195 | 0.00043 | 0.16960 | 1.09 |
| ChE(18:2) | 0.02343 | 0.00987 | 0.02023 | 0.00789 | 0.17235 | 1.16 |
| Cer(d18:1/24:1) | 0.00010 | 0.00005 | 0.00010 | 0.00009 | 0.18080 | 1.01 |
| ChE(18:1) | 0.00746 | 0.00223 | 0.00675 | 0.00224 | 0.18369 | 1.11 |
| PC(36:3) | 0.01029 | 0.00279 | 0.00927 | 0.00255 | 0.18369 | 1.11 |
| PC(38:5) | 0.00711 | 0.00176 | 0.00648 | 0.00155 | 0.20172 | 1.10 |
| MePC(37:3) | 0.00026 | 0.00010 | 0.00024 | 0.00010 | 0.21982 | 1.13 |
| PC(40:6p) *1* | 0.00027 | 0.00007 | 0.00025 | 0.00009 | 0.23459 | 1.09 |
| SM(d36:2) | 0.00529 | 0.00096 | 0.00505 | 0.00111 | 0.23459 | 1.05 |
| PC(40:6) | 0.00693 | 0.00208 | 0.00633 | 0.00183 | 0.23808 | 1.09 |
| PC(38:6) | 0.00882 | 0.00258 | 0.00800 | 0.00195 | 0.24160 | 1.10 |
| TG(18:0/16:0/18:1) | 0.03358 | 0.01743 | 0.03881 | 0.02225 | 0.26349 | 0.87 |
| MePC(37:3p) *1* | 0.00015 | 0.00010 | 0.00014 | 0.00010 | 0.30295 | 1.09 |
| MePC(38:2e) | 0.00012 | 0.00005 | 0.00011 | 0.00003 | 0.32442 | 1.12 |
| MePC(38:1) | 0.00077 | 0.00032 | 0.00071 | 0.00030 | 0.33280 | 1.09 |
| MePC(35:0p) | 0.00109 | 0.00072 | 0.00097 | 0.00074 | 0.35984 | 1.12 |
| PC(40:4) | 0.00136 | 0.00031 | 0.00130 | 0.00037 | 0.38822 | 1.04 |
| PC(16:0/18:1) | 0.27126 | 0.06187 | 0.25749 | 0.06871 | 0.43327 | 1.05 |
| SM(d36:0) | 0.00042 | 0.00011 | 0.00040 | 0.00011 | 0.43327 | 1.04 |
| SM(d32:1) | 0.00169 | 0.00022 | 0.00165 | 0.00036 | 0.45956 | 1.02 |
| PC(36:5) | 0.00079 | 0.00050 | 0.00071 | 0.00041 | 0.46492 | 1.10 |
| PI(18:0/20:4) | 0.00105 | 0.00025 | 0.00104 | 0.00033 | 0.49781 | 1.01 |
| PE(16:0p/20:4) | 0.00058 | 0.00020 | 0.00054 | 0.00016 | 0.50904 | 1.08 |
| PC(35:4) | 0.00052 | 0.00012 | 0.00054 | 0.00018 | 0.51470 | 0.95 |
| MePC(39:1p) | 0.00008 | 0.00005 | 0.00009 | 0.00014 | 0.53897 | 0.82 |
| ChE(22:6) | 0.00525 | 0.00215 | 0.00494 | 0.00166 | 0.56712 | 1.06 |
| PC(30:0) | 0.00596 | 0.00113 | 0.00600 | 0.00128 | 0.59122 | 0.99 |
| MePC(37:1p) | 0.00012 | 0.00007 | 0.00012 | 0.00011 | 0.59696 | 1.03 |
| PC(37:5) | 0.00021 | 0.00008 | 0.00021 | 0.00009 | 0.67258 | 1.02 |
| PC(31:0) | 0.00110 | 0.00019 | 0.00107 | 0.00023 | 0.73132 | 1.03 |
| ChE(20:5) | 0.00299 | 0.00207 | 0.00272 | 0.00171 | 0.75127 | 1.10 |
| MePC(33:1) | 0.01973 | 0.00803 | 0.02085 | 0.01013 | 0.75127 | 0.95 |
| SM(d36:1) | 0.01822 | 0.00365 | 0.01821 | 0.00504 | 0.75796 | 1.00 |
| SM(d43:2) | 0.00014 | 0.00007 | 0.00015 | 0.00010 | 0.79168 | 0.95 |
| PC(33:0) | 0.00081 | 0.00017 | 0.00080 | 0.00023 | 0.83265 | 1.01 |
| PC(11:0/21:0) | 0.03634 | 0.00697 | 0.03631 | 0.00794 | 0.86715 | 1.00 |
| PC(32:2) | 0.00008 | 0.00004 | 0.00009 | 0.00005 | 0.86955 | 0.95 |
| SM(d34:2) | 0.00227 | 0.00036 | 0.00223 | 0.00047 | 0.87408 | 1.01 |
| MePC(32:1) | 0.00028 | 0.00020 | 0.00028 | 0.00021 | 0.88235 | 0.98 |
| MePC(37:1) | 0.00093 | 0.00053 | 0.00094 | 0.00052 | 0.91586 | 1.00 |
| PE(16:0p/22:6) | 0.00103 | 0.00028 | 0.00103 | 0.00032 | 0.98595 | 1.00 |
| PE(18:0p/22:6) | 0.00365 | 0.00110 | 0.00365 | 0.00125 | 0.98595 | 1.00 |
| PC(37:6) | 0.00167 | 0.00052 | 0.00167 | 0.00062 | 0.99298 | 1.00 |

- **Supplementary Figure S1**: Venn diagram constructed for the lipids highlighted by univariate analysis (Wilcoxon test by Metaboanalyst *via* the volcano plot, and with the Benjamini-Hochberg correction) and multivariate analysis (OPLS-DA using SIMCA®) to discriminate between patients with ALS and controls (n=85). Lipids marked with an asterisk (*) were also highlighted by the *biosigner* analysis. All the discriminant lipids were at significantly lower levels in cases of ALS than in controls, except for TG (16:1/18:1/18:2).


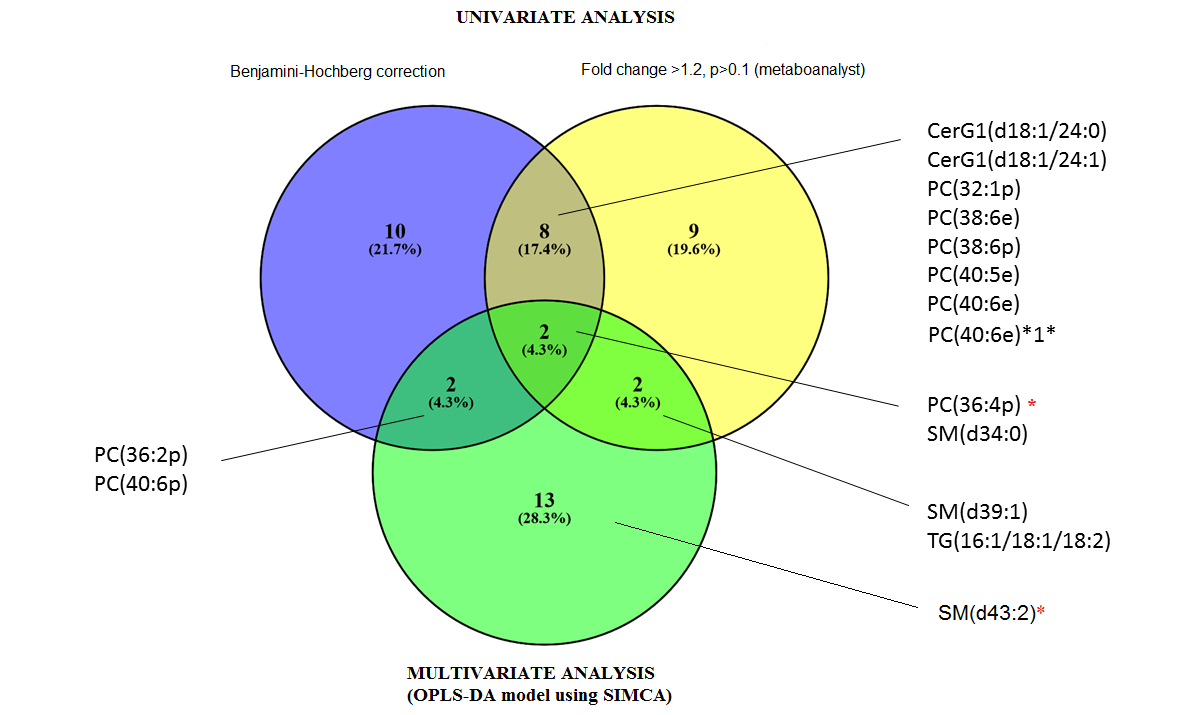

Supplement: Supplementary file 1 — supplementary information [file 41598_2017_17389_MOESM1_ESM.doc]
